# Clarifying Karma for Culturally Concordant Care

**DOI:** 10.1200/GO.23.00259

**Published:** 2023-10-19

**Authors:** Rushil V. Patel, Komal P. Murali, Viraj R. Patel

**Affiliations:** Rushil V. Patel, MD, Division of Hematology and Oncology, University of Alabama at Birmingham (UAB), Birmingham, AL; Komal P. Murali, PhD, RN, ACNP-BC, New York University Rory Meyers College of Nursing, New York, NY; and Viraj R. Patel, MPH, Rush University Medical Center, Chicago, IL

## TO THE EDITOR:

We thank the authors for this timely review highlighting how culture influences the communication preferences for Asian patients with cancer.^1^ As they note, religious beliefs affect these considerations, and they recommend clinicians use religious terminology to demonstrate their understanding of Hindu beliefs. However, we hope to provide a more nuanced understanding of the belief in karma in contrast to the authors' presentation of this concept, which we feel may inadvertently propagate the notion that Hindu patients may prefer suffering over symptom management at the end of life.

Karma, or action, is the principle of cause and effect where actions produce consequences across multiple rebirths (ie, reincarnation), and the aim of life for Hindus is to liberate oneself from this karmically induced cycle of rebirth (moksha).^1^ While specific understanding of karmic consequences varies significantly across and within religions (eg, Hinduism, Buddhism, Jainism, and Sikhism), most agree that the intent and performance of an action are governed by free will. The process by which karmic consequences manifest across lifetimes is, however, generally beyond human perception or control. Therefore, the doctrines of karma and rebirth emphasize positive future action as a means to correct the past, not the endurance of suffering. In the context of serious illness, while some may interpret their illness as a consequence of negative past actions, greater weight would likely be placed on reducing symptoms to continue karmically beneficial activities important to one's values, such as prayer or charity.^2^ According to this framework, few Hindus would argue that foregoing symptom management produces a longitudinal karmic benefit, as has been implied in case reports^3^ and literature reviews,^4^ which have concluded that end-of-life comfort is only concordant with Christianity and Judaism but not with Hinduism or Buddhism.^5^ We worry that this misunderstanding may widen inequities in care, especially when previous studies of Asian patients in the United States,^6^ Canada,^7^ and India^8^ have conversely demonstrated how the belief in karma can coexist with symptom management.

We agree with the authors' recommendation for future research exploring how religious beliefs influence preferences for serious illness communication. Noted in ASCO guidelines for addressing disparities to achieve health equity,^9^ community-based participatory research (CBPR) is an approach rooted in social justice, whereby investigators engage the community as an equal partner to develop solutions for improving health outcomes and addressing disparities.^10^ Investigators first convene a community advisory group (CAG), which includes community leaders and other members who have experiences with the topic of interest. Researchers then meet with the CAG to review each aspect of the proposed study to incorporate their insights, even changing direction if needed. For example, if investigators plan to conduct focus group meetings, the CAG may identify factors (eg, gender or age dynamics) that might influence the discussions, allowing the investigators to modify the design to maximize input. Finally, all knowledge generated through this partnership belongs to the community. Investigators thus present the final results back to the CAG and other community members who participated in the study to ensure the results are aligned with the community's experiences before dissemination.

One author (R.V.P.) is conducting a CBPR project exploring how culture influences the palliative care preferences of Hindu patients with cancer and caregivers living in Charlotte, North Carolina.^11^ In reviewing the focus group design, CAG members recommended not to exclude participants with limited English proficiency (LEP). They felt that these individuals experienced unique challenges having to rely on others to facilitate communication at their oncology visits. However, the CAG members also acknowledged the linguistic diversity of this community and the difficulty identifying interpreters proficient in each language. For example, India, home to the largest Hindu community, has 22 national languages, including English. The CAG members suggested a smaller focus group size, so participants with LEP can identify another who can facilitate their participation in the focus group, like the individual who accompanied them to their appointments. Such considerations are best highlighted by community members—the hallmark of CBPR.

We applaud the authors for highlighting these considerations, given the challenge of summarizing the literature pertaining to diverse communities. We thus clarify that the belief in karma does not exclude symptom management; CBPR can further identify these nuances to realize culturally concordant care everywhere.
